# Considerations for FAIR data catalogs from DAPHNE4NFDI

**DOI:** 10.1107/S2052252526006871

**Published:** 2026-08-06

**Authors:** Bishoy Hakim, Fabio Dall’Antonia, Johannes Dallmann, Christian Felder, Abhijeet Gaur, Heike Görzig, Nicolas Hayen, Regina Hinzmann, Julia Kobus, Rolf Krahl, Bridget Murphy, Sebastian Paripsa, Björn Pedersen, Agha Raza, Kristin Tippey, Tim Wetzel, Alexander Zaft, Tobias Unruh, Sebastian Busch

**Affiliations:** a Institute for Crystallography and Structural Physics (ICSP), Friedrich–Alexander-Universität Erlangen–Nürnberg (FAU), Germany; b European XFEL GmbH (EuXFEL) Germany; c Jülich Centre for Neutron Science (JCNS) at Heinz Maier-Leibnitz Zentrum (MLZ), Forschungszentrum Jülich GmbH (FZJ), Germany; d Institute of Technical Chemistry and Polymer Chemistry, Karlsruhe Institute of Technology (KIT), Germany; e Helmholtz-Zentrum Berlin für Materialien und Energie (HZB) Germany; f Institute of Experimental and Applied Physics Kiel University (CAU) Germany; g Deutsches Elektronen-Synchrotron (DESY), Germany; h Ruprecht Haensel Laboratory CXNS Deutsches Elektronen-Synchrotron DESY Germany; i Condensed Matter and Experimental Solid State Physics, University of Wuppertal (BUW), Germany; j Technical University of Munich (TUM) at Heinz Maier-Leibnitz Zentrum (MLZ), Germany; k Universität Siegen, Germany; l Helmholtz-Zentrum Dresden–Rossendorf (HZDR), Germany; m German Engineering Materials Science Centre (GEMS) at Heinz Maier-Leibnitz Zentrum (MLZ) Helmholtz-Zentrum Hereon GmbH (Hereon) Germany; UCL, United Kingdom

**Keywords:** FAIR data principles, metadata catalogs, research data management, persistent identifiers, photon and neutron science

## Abstract

Large-scale photon and neutron (PaN) experiments generate vast amounts of valuable data, yet implementation of the FAIR principles (findable, accessible, interoperable and reusable) remains incomplete. Metadata catalogs are a key tool for addressing this challenge. They do not store data itself but provide structured searchable descriptions that make datasets discoverable and reusable. This article, developed within the DAPHNE4NFDI consortium of Germany’s National Research Data Infrastructure (NFDI), reflects the discussion on the role of metadata catalogs in PaN science such as SciCat, ICAT and SampleDB from a scientific point of view, and presents the status of current implementations. Integration of standards such as NeXus, sample databases and electronic laboratory notebooks is also addressed. Catalog implementations are categorized by purpose as raw data, institutional data and public data, and examined by practical aspects including authentication and authorization, persistent identifiers, and metadata enrichment. Drawing on case studies such as the RefXAS X-ray absorption spectroscopy database, we summarize lessons learned and present recommendations for minimizing fragmentation, enabling interoperability, and harmonizing identity and persistent-identifier policies. Looking ahead, semantic technologies and knowledge graphs will play a central role in building a federated FAIR-compliant catalog infrastructure that supports both facility and university researchers.

## Abstract 2

Metadata catalogs are essential for implementing FAIR data principles in photon and neutron (PaN) science, enabling experimental data to become findable, accessible, interoperable and reusable. This work provides an analysis of current catalog systems in the PaN field and offers practical recommendations to advance interoperability and build a federated FAIR-compliant data infrastructure.

## 1. Introduction

Experiments at large-scale photon and neutron (PaN) facilities generate enormous volumes of valuable data. Without structured metadata and catalog systems, much of these data remain difficult to find and reuse. The FAIR principles (findable, accessible, interoperable and reusable) (Wilkinson *et al.*, 2016 ▸) provide a framework for addressing these challenges, but their implementation requires practical tools. Metadata catalogs are one such tool: they do not store the data itself but provide searchable descriptions that make datasets discoverable and accessible.

Following the rapid growth of scientific data, the adoption of FAIR principles is a focus of current work. Enabling efficient data exchange and reuse is essential for accelerating research and ensuring long-term value. This requires more than standardized file formats; it demands systems that support the full data lifecycle from creation and processing to curation and publication while preserving provenance and enabling interoperability.

The objective of this article is to explain the role of metadata catalogs in FAIR data management, compare leading catalog systems and share lessons learned from their deployment. We focus on solutions relevant to the PaN science community, drawing on use cases from the DAPHNE4NFDI project (Barty *et al.*, 2023 ▸). Our discussion includes conceptual foundations, technical approaches, and practical experiences from a consortium of major German facilities and universities working together to advance catalog adoption in PaN sciences.

## 2. Conceptual framework

### 2.1. FAIR principles and their relevance

The FAIR principles provide a widely accepted framework for improving the value and usability of research data. For scientists working at large-scale facilities or in university laboratories, these principles mean that data should not only be preserved but also easy to locate and accompanied by sufficient context for meaningful interpretation.

In practice, ‘findable’ implies that datasets are assigned globally unique and persistent identifiers (PIDs), such as digital object identifiers (DOIs), and described with rich metadata so that both humans and machines can discover them efficiently. Crucially, this discovery works via the catalogs described in this contribution. ‘Accessible’ means that data and metadata can be retrieved through standardized protocols, with clear conditions for access, including authentication and authorization where necessary. ‘Interoperable’ requires the use of community standards for formats and vocabularies, such as NeXus (Könnecke *et al.*, 2015 ▸) (https://www.nexusformat.org/) in PaN science, so that data can be integrated and exchanged across different systems and disciplines. Finally, ‘reusable’ emphasizes accurate provenance and adherence to established standards, enabling replication and further analysis under transparent licensing terms.

Implementing these principles ensures that research outputs are optimized for transparency, reproducibility and long-term impact. While the original definitions focused on machine readability and automation (Wilkinson *et al.*, 2016 ▸), they are equally relevant for improving how scientists interact with data in practice (Vogt *et al.*, 2025 ▸).

### 2.2. Role of metadata catalogs

FAIR data require that datasets are also easy to find and access. This is achieved through metadata catalogs, which provide structured descriptions (the metadata) of data and references to the actual files. A useful analogy is library catalogs: they provide an inventory of the library and allow searching for the books based on bibliographic metadata, such as author, title and publisher. These catalogs also provide information about where the book can be found in the library. But they do not contain the books themselves. Similarly, a scientific data catalog does not contain the datasets, but offers enough information for users to identify what they need and decide whether to obtain it.

Historically, PaN data catalogs have often been limited to barely documented raw data, with a few exceptions in specialized fields such as the Protein Data Bank (Berman *et al.*, 2003 ▸) or Coherent X-ray Imaging Data Bank (Maia, 2012 ▸). Efficient search features and suitable facility data policies are not always implemented. For many users at large-scale PaN facilities, even having raw data conveniently accessible for download after an experiment is a significant improvement. However, FAIR catalogs go beyond this basic functionality.

The difference between a FAIR catalog and a simple file explorer lies in the richness and accessibility of metadata. A file explorer can show file names, sizes and creation dates, but little else. A catalog, by contrast, can include experimental details such as sample temperature or instrument configuration. These details are extracted from the data files or recorded during ingestion, the process of adding a dataset to the catalog. Moreover, catalogs maintain an index of all entries in a database, enabling fast searches and filtering by any indexed metadata field – something a file explorer cannot do efficiently. There are different ways to implement such indexing. One approach uses relational databases, as in the ICAT system (https://icatproject.org/). Here, the structure of the database is defined in advance, and every entry must provide values for the same set of fields. This ensures consistency and makes searching straightforward, but it limits flexibility: adding new unforeseen metadata fields is difficult. The alternative approach, used by SciCat (https://scicatproject.org/) (https://github.com/ScicatProject), relies on a document-based NoSQL database (Trageser *et al.*, 2025 ▸). Each entry can include its own set of metadata, allowing adaptation to diverse experimental needs. This flexibility is attractive but comes with the risk of inconsistency if standards are not followed, making comparisons and filtering harder. In this case, responsibility lies with the data ingestor to maintain agreed conventions.

Both approaches have clear merits. ICAT’s structured design supports predictable workflows and robust querying, which is valuable for facilities with well defined processes. SciCat’s schema-free model offers adaptability for evolving experiments and currently enjoys strong momentum within the PaN community. Choosing between them depends on institutional priorities and the balance between flexibility and standardization.

### 2.3. Catalog users and their requirements

From a user perspective, data catalogs will increasingly act as the central entry point connecting proposal information, sample metadata, experimental conditions, raw data and downstream analysis results throughout the full experimental lifecycle. Already during the experiment, the catalog gives on-site and remote users a convenient overview of the acquired datasets. It serves as a starting point to launch Jupyter Notebook (https://jupyter-notebook.readthedocs.io/) or other analysis programs in which the selected data are automatically mounted. After the experiment, researchers can search and sort their data in the catalog using sample identifiers, proposal numbers, chemical composition, instrument parameters, or derived metadata in order to retrieve raw or processed datasets for further analysis or reuse. If a suitable collection of data has been assembled, a DOI minting process can be launched from the catalog that allows identification of this particular set of data. Later in time (after an embargo period), even the data of other research projects can be accessed and presented in an understandable way.

Metadata catalogs must therefore serve a diverse community of scientists, each with different needs and expectations. At large-scale facilities, non-expert users as well as expert instrument scientists require access to raw data and technical metadata during and soon after an experiment. This information supports immediate collaboration and troubleshooting during beam time and in the days following data collection.

External researchers, including future collaborators or scientists from other institutions, often focus on processed and curated or published datasets rather than raw instrument output. Their interest lies in higher-level metadata that describes the scientific context of the data (such as sample composition, experimental method and analysis results) rather than technical details of the beamline.

Between these two extremes are processed or reduced datasets that result from automated workflows or standardized transformations. These are particularly relevant for big data applications, where well tagged data are needed. In such cases, catalogs must allow users to add custom tags or annotations to facilitate reuse.

The requirements for catalogs therefore vary across user groups. Some users need rapid access to raw data for quality checks, while others prioritize discoverability of curated datasets for long-term reuse. A well designed catalog landscape must accommodate this spectrum by supporting flexible metadata structures, efficient search capabilities and clear access-control policies. It should also maintain links between raw, processed, and published data to preserve provenance and enable reproducibility.

### 2.4. Types of metadata catalogs and their roles

The requirements for the metadata catalogs defined above are not all the same; they serve different purposes depending on the stage of the data lifecycle and the intended audience. Within DAPHNE4NFDI, three main categories have been identified.

First, raw-data catalogs are automatically populated by beamlines or laboratory instruments with minimal user interaction. They provide immediate access to experimental metadata for facility users and instrument scientists, typically under strict access control and hosted at the data-taking institute. These catalogs can also contain information about processed or reduced datasets that result from automated workflows or standardized transformations, since these are often run at the large-scale facility.

Second, internal catalogs support research groups at universities or research institutes. These catalogs combine data collected by a certain research group at local instruments and large-scale facilities, enabling unified access and search capabilities within the group. Like raw-data catalogs, they usually enforce stringent access rights.

Third, public data catalogs are designed for curated datasets that have undergone quality checks and curation. These catalogs allow authenticated users to contribute data, while read access is open to the broader community. Public catalogs are often hosted centrally to serve the entire German PaN community and, eventually, international users.

In addition to these core types, related systems play an important role. Sample catalogs such as SEPIA (Krahl *et al.*, 2024 ▸; Sedeqi *et al.*, 2025 ▸) (https://codebase.helmholtz.cloud/hzb/research_data_management/sepia) at HZB record detailed information about samples, including their history and properties, which is usually essential for interpreting experimental results. Publication catalogs track outputs such as journal articles, data publications, patents and outreach activities. Integration between all of these catalogs, and also electronic laboratory notebooks (ELNs) (Jordt *et al.*, 2024 ▸), is crucial for maintaining provenance and enabling data reuse. PIDs for samples (*e.g.* International Generic Sample Number – IGSN) (Klump *et al.*, 2021 ▸) (https://igsn.uni-kiel.de/) and datasets ensure that these links remain stable over time.

These distinctions matter because each catalog type addresses different user needs: rapid access to raw data for facility scientists, organized storage for research groups, and open sharing of datasets for the wider community. A coherent strategy that connects these systems will maximize the value of PaN data and support FAIR principles. The distinction does not necessarily imply that the catalogs have to be run as independent software systems, even though this is often the case.

### 2.5. How much and which metadata should be in the catalog?

A key question in designing a metadata catalog is how much information should be included. This discussion is not about what is recorded during the experiment – community standards such as NeXus define that – but about which details should be stored in the catalog to enable efficient search so that the NeXus file with all the details about the experiment can be found in the first place. The answer depends on user needs, which vary widely. An instrument scientist might want to review typical configurations used in past experiments. A domain specialist may search for data on a specific sample, while a big data analyst might look for correlations between parameters across many datasets. No single solution can satisfy all possible queries, but three general approaches have emerged in the community.

The first approach focuses on the findable aspect of FAIR. Catalogs following this philosophy include only the metadata necessary for typical searches, such as experiment date, instrument and sample name. This keeps the catalog lean and easy to maintain.

The second approach assumes that database performance is sufficient and ingests all available metadata into the catalog. In this case, the catalog mirrors the metadata stored in the data files, providing maximum flexibility for future queries.

The third approach addresses scenarios where raw-data files become inaccessible after archiving. Here, the catalog not only stores metadata from the data files but also allows enrichment after ingestion. This enables users to correct errors or add information such as quality indicators at a later stage, when the original files cannot be modified.

Each approach has advantages and trade-offs. Capturing only essential metadata simplifies indexing and search but limits future possibilities. Collecting everything ensures completeness but may lead to complexity and higher maintenance. Allowing enrichment supports evolving workflows but requires safeguards to prevent unintended changes. A mechanism has to be implemented that documents these changes to the metadata parameters in the catalog. This would allow one to create a list of who changed which parameter when and why.

A practical compromise is to define a core set of indexed metadata fields that support common search scenarios, while permitting additional fields for subject-specific needs. Catalogs should also provide mechanisms for metadata updates after ingestion, without altering the original raw data files. Provisions to reconcile this additional or updated information with the information in the files when requested for download have to be made.

Integration with ELNs and sample databases can further enhance context and usability. Ultimately, the goal is to balance completeness with usability, ensuring that catalogs remain effective tools for finding and reusing scientific data.

### 2.6. Persistent identifiers and their role

PIDs are essential for ensuring that datasets, samples, and related research outputs remain uniquely identifiable and accessible over time. They provide stable links that do not change even if the underlying storage or catalog system does, which is critical for implementing FAIR principles. These links lead to ‘landing pages’, where the most important metadata information of the described dataset is summarized; this page can be, but does not have to be, generated by a catalog.

Several PID systems are in use. DOIs (https://www.doi.org/the-identifier/what-is-a-doi/) are widely recognized and com­monly used for publications and curated datasets. They include metadata fields such as author names, which can be challenging for beamline data because the individuals performing specific measurements are often not recorded. For this reason, some facilities, such as HZB, use handles that do not require author information for raw data. For samples, the IGSN system provides globally unique identifiers and is already used by institutions such as Kiel University.

It is important to distinguish between published data and public data. While a large portion of facility data may eventually become publicly accessible, it does not automatically qualify as published. Only datasets referenced in scientific publications and explicitly flagged for publication typically receive a DOI, signaling their formal recognition as part of the scholarly record. The publication process often includes the curation of bibliographic metadata. Other datasets may remain public without being formally published.

For internal workflows, especially at universities, assigning PIDs to raw data can still be valuable even if the data are not destined for publication. PIDs enable reliable linking between raw, processed and curated datasets, as well as between data and associated samples or publications. This linkage supports provenance tracking and long-term reuse, which are central goals of FAIR data management. Assigning PIDs to datasets is therefore a prerequisite for FAIR data.

## 3. Technical solutions

### 3.1. General architecture of a catalog system

**Figure 1:**
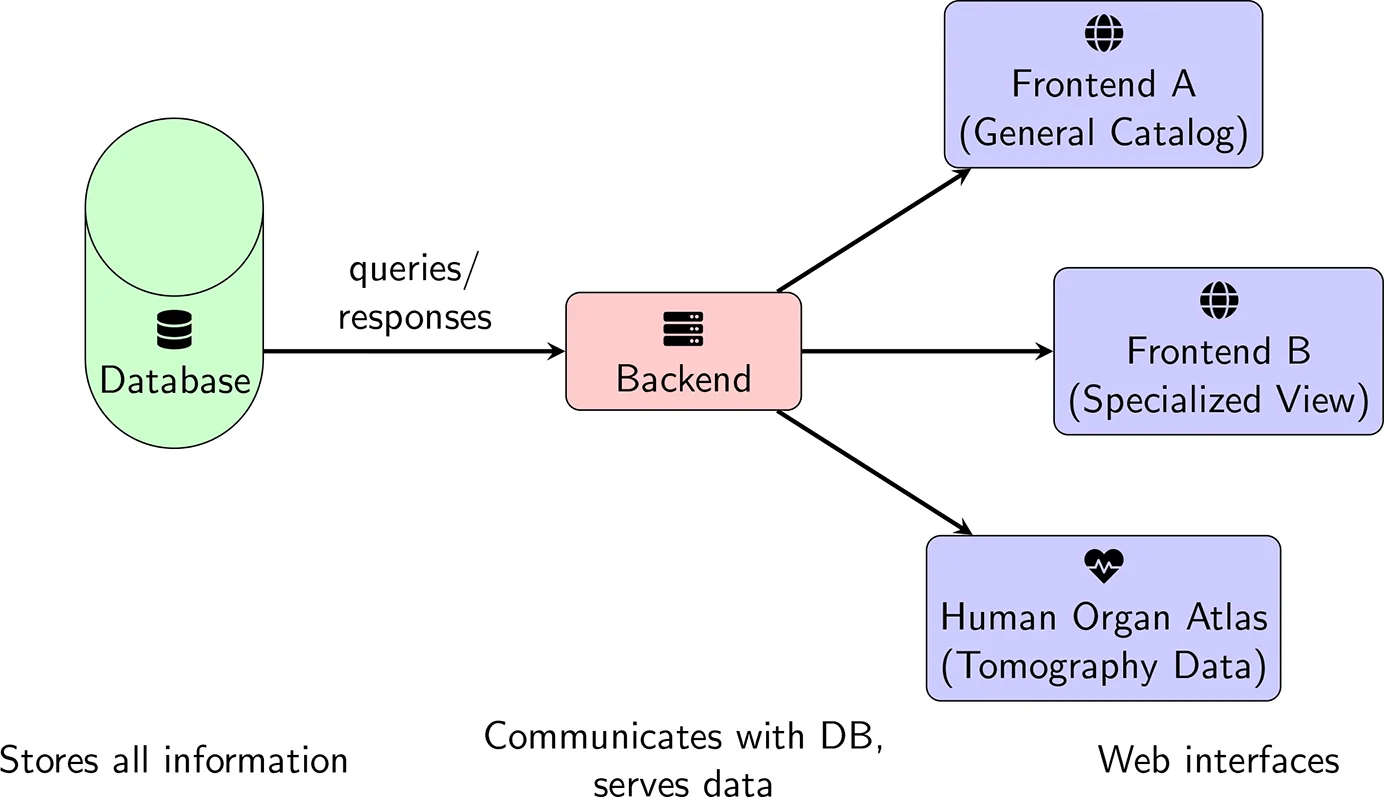
Overview of the interplay between database (DB), backend and frontend in a typical metadata catalog.

The catalog programs pertinent to this contribution are organized in the way summarized in Fig. 1 ▸: a database contains all the information; and a piece of software called the backend communicates with this database, sending the right queries. It sends the responses of the database to the frontend, which is essentially a website that displays the responses conveniently. There can be several frontends corresponding with the same backend if one wants to have a particular view on the data. For example, the human-organ atlas (https://human-organ-atlas.esrf.fr/) is a specialized frontend of the ESRF open-data catalog (https://www.esrf.fr/ICAT), which only displays tomography data of human organs and, due to this specialization, can provide a look and feel that is extremely useful for these data but would be inadequate for other types of measurements.

### 3.2. Overview of catalog systems in PaN science

Unlike the web, research data are managed through multiple independent catalog platforms. Each system is designed for specific communities or data types, and there is no universal catalog that contains everything. As a result, scientists often navigate across different platforms to find and manage their data.

Among the catalog systems used in PaN science, two play a central role: ICAT and SciCat. ICAT is based on a relational database model for administrative/bibliographic metadata, where the structure of the metadata is defined in advance. This approach enforces consistency and supports powerful query capabilities, making it well suited for facilities with established workflows and strict data policies. Physical metadata definitions are globally managed as a list of allowed metadata and respective units. This list applies to the whole catalog and therefore supports facility-wide searches on standardized metadata.

SciCat, in contrast, uses a document-based NoSQL database. This schema-free design allows each dataset to include its own set of metadata, offering adaptability for diverse experiments and evolving requirements. The trade-off is that flexibility can lead to inconsistency if standards are not maintained. SciCat is a younger system that has gained strong momentum over the past few years in the PaN community, partly because of its openness and active development.

Other systems address complementary needs. SampleDB (Rhiem, 2021 ▸) (https://scientific-it-systems.iffgit.fz-juelich.de/SampleDB/index.html) focuses on sample-centric metadata and supports federation across facilities, enabling researchers to track samples measured at different instruments. NOMAD Oasis (Draxl & Scheffler, 2018 ▸) (https://nomad-lab.eu/nomad-lab/), developed within the FAIRmat consortium, provides a broader platform for managing data from condensed matter physics, including a metadata catalog integrated with analysis workflows.

Each system has its merits: ICAT offers stability and predictable structure, while SciCat provides adaptability and community-driven innovation. SampleDB and NOMAD Oasis extend functionality beyond experiment catalogs, linking samples and computational data. The coexistence of these systems underscores the importance of interoperability and common standards to ensure that data remain findable and reusable across institutional and national boundaries, especially when thinking of searches in federated catalog environments (*cf*. Section 4.2).

Although many of the examples discussed in this work originate from Europe-based initiatives such as PaNOSC, ExPaNDS and DAPHNE4NFDI, comparable developments are also ongoing internationally, where similar pressures arising from rapidly increasing data volumes and the need for reproducibility have led to widespread recognition of FAIR-aligned practices. Synchrotron and neutron facilities across the world face analogous challenges, including the management of large data streams, metadata standardization, and the integration of data-analysis workflows into experimental environments. The technical solutions, such as the NeXus data format, are developed through international collaboration, so they already provide a shared foundation for interoperability across facilities worldwide.

The implementation of FAIR principles is however heterogeneous and often driven by individual facilities or national laboratory systems rather than by coordinated cross-facility consortia. The most significant difference therefore lies not in the underlying trends but in the mode of organization and governance. European initiatives explicitly frame FAIR data as a collective endeavor requiring harmonization across infrastructures, supported by dedicated projects and policy frameworks. This has led to the development of common metadata models, federated catalogs and shared data policies, albeit still with substantial diversity at the implementation level. In contrast, North American and Australasian efforts tend to prioritize robust local data ecosystems embedded in national funding structures, with cross-facility interoperability emerging more gradually and often through *de facto* standards rather than formal coordination.

## 4. Implementation landscape

Most institutions now operate some form of catalog, with SciCat widely adopted and ICAT remaining in use at major facilities. While practices differ, the majority favor SciCat for new deployments, with notable exceptions where ICAT’s maturity and stability are valued, such as at HZB or ESRF. DOI minting and interoperability standards like the PaNOSC search application programming interface (API) and the Open Archives Initiative Protocol for Metadata Harvesting (OAI-PMH) (Bodera *et al.*, 2023 ▸; Minotti & Servan, 2023 ▸) are increasingly implemented, although progress varies.

### 4.1. Institutional status quo

Across the German PaN community, several catalog systems are in active use (*cf*. Table 1 ▸), reflecting different institutional priorities and legacy infrastructures. While SciCat has been recommended by DAPHNE4NFDI for new implementations, ICAT remains well established at major facilities, and complementary systems such as SampleDB and NOMAD Oasis address specific needs.

#### 4.1.1. SciCat

SciCat is increasingly adopted by facilities and universities due to its flexibility and active development. Institutions such as DESY, MLZ, HZDR, ESS and Kiel University have committed to SciCat for raw-data catalogs and, in some cases, for public data portals. DESY recently switched from operation of multiple SciCat instances to a more centralized service still within a Kubernetes environment, while MLZ plans a centralized SciCat deployment to serve multiple instruments. FAU will use SciCat for public data access alongside other tools. This trend reflects a community-wide effort to consolidate around a system that supports diverse metadata and future interoperability.

The ingestion of information into SciCat happens through one of two routes. The first option, followed *e.g.* at FAU, is that NeXus files are read by the ingestor and pertinent information is extracted. The second possibility, in use for example at MLZ, is that the instrument-control software produces event triggers (webhook, Kafka, RabbitMQ) that reach the ingestor in a data stream that runs independently from the writing of the data files.

#### 4.1.2. ICAT

ICAT remains the backbone of data cataloging at facilities with mature workflows. HZB uses ICAT as its central metadata catalog (including both raw data and data publications), integrating access control and automated staging from tape to disk. Other major European facilities, including ESRF, ISIS, ALBA and Diamond, also rely on ICAT, underscoring its stability and proven track record. ICAT has been proven to scale to large data volumes: Diamond has more than two billion datafiles in its catalog. While ICAT’s rigidity limits flexibility, its structured approach simplifies complex queries and supports robust data-management policies.

#### 4.1.3. SampleDB

SampleDB is used at FZJ for managing sample-centric metadata. Its schema-based design ensures high-quality metadata and supports federation across facilities, enabling researchers to track samples measured at different instruments. SampleDB complements SciCat by providing detailed sample information and offers export capabilities for integration with other catalogs.

#### 4.1.4. NOMAD

NOMAD Oasis, developed within the FAIRmat consortium, is gaining visibility at FAU and other partners. It provides a comprehensive platform for managing data from condensed matter physics, including a metadata catalog integrated with analysis workflows. NOMAD’s adoption reflects growing interest in linking experimental and computational data under FAIR principles.

#### 4.1.5. Invenio

At HZDR, Invenio (https://invenio-software.org) (https://github.com/inveniosoftware/awesome-invenio) is used alongside SciCat and HELIPORT (https://codebase.helmholtz.cloud/heliport/heliport) for workflow tracking and data publication. Invenio’s role is primarily in managing curated datasets and supporting open-data consortiums.

### 4.2. Interoperability and federation

For FAIR principles to be fully realized, metadata catalogs must not operate in isolation. Interoperability enables researchers to discover data across facilities and integrate them into broader scientific workflows. Two key mechanisms support this goal: the PaNOSC (https://www.panosc.eu/) search API (https://data.panosc.eu/) and the OAI-PMH protocol (https://www.openarchives.org/pmh/). PaNOSC endpoints provide a standardized interface for federated searches across PaN facilities, while OAI-PMH allows metadata harvesting by external services such as B2FIND (https://b2find.eudat.eu/). All catalogs relevant for DAPHNE4NFDI should include the PaNOSC search capabilities, and OAI-PMH endpoints are strongly recommended.

Several institutions have already implemented these interfaces. HZB offers an OAI-PMH endpoint that is regularly harvested by B2FIND. Published DESY data are being harvested using the SciCat PaNOSC search API and the OAI-PMH endpoints in the PaN Finder (https://pan-finder.panosc.ess.eu/). MLZ plans to include both the PaNOSC search API and OAI-PMH in its SciCat deployment, while Kiel University intends to integrate PaNOSC endpoints in its upcoming catalog.

These efforts reflect a growing recognition that interoperability is essential for building a federated catalog ecosystem. By adopting common APIs and protocols, facilities can ensure that their data are not only findable locally but also discoverable across the PaN community and beyond.

### 4.3. Authentication and authorization infrastructure

Managing user identities and access rights is a critical aspect of metadata-catalog deployment. Authentication ensures that users can log in securely, while authorization defines what actions they are permitted to perform – such as viewing, uploading or curating data. Authentication and authorization infrastructure (AAI) is therefore essential for implementing FAIR principles in practice, particularly for catalogs that span multiple institutions.

Several approaches are currently in use across the PaN community. DESY employs a federated login system based on Keycloak (https://github.com/keycloak/keycloak), integrated with Helmholtz ID as an external identity provider, and plans to link this with its proposal system. FAU relies on internal accounts for authentication, while FZJ and KIT are moving toward Helmholtz ID for both internal and external users. HZB is developing a central identity management system to unify access across its services, including catalogs, storage systems and ELNs.

Federated identity providers like Helmholtz ID (https://hifis.net/aai/), umbrellaID (https://umbrellaid.org/) and ORCID (https://orcid.org/) offer significant advantages for mobility and collaboration. Scientists frequently move between institutions, and a common AAI framework ensures continuity of access without duplicating user accounts. However, practical challenges remain, such as mapping roles across systems and maintaining consistent user identifiers when affiliations change.

A harmonized approach to AAI such as pursued in the frame of Base4NFDI (https://base4nfdi.de/) will be essential for building a federated catalog ecosystem. By adopting shared identity providers and a common role model that covers administrators, instrument scientists, data curators and external users, the PaN community can enable secure seamless access to data while supporting interoperability across facilities.

### 4.4. Persistent-identifier workflows for published data

Using PIDs such as ORCID for person identification and ROR for organization identification (https://ror.org/) along with assigning PIDs such as DOIs to datasets is a key step in making research data citable and reusable (Juty *et al.*, 2020 ▸). DOIs provide a stable reference that can be included in publications, ensuring that data remain accessible and linked to its scientific context. However, practices for DOI minting vary significantly across institutions.

DESY has introduced a DOI minting service where selected datasets can be published (Kwee-Hinzmann *et al.*, 2026 ▸). HZB follows a different approach, reserving DOIs exclusively for curated data publications rather than raw data. Raw data get ePIC handles. FAU and CAU prefer to mint DOIs when a dataset becomes part of a publication, but a formal standard for this process is still under discussion.

These variations reflect differing interpretations of what constitute publishable data and how identifiers should be managed. While some institutions treat all beam-time data as potentially publishable, others restrict DOI minting to curated datasets referenced in scientific literature. Harmonizing these workflows across the PaN community would improve consistency, facilitate data citation and strengthen compliance with FAIR principles.

### 4.5. Sample databases and their integration with catalogs

In PaN research, understanding the history and properties of a sample is often essential for interpreting experimental results. While metadata catalogs describe datasets, they typically do not capture the full lifecycle of a sample – from preparation and characterization to measurement and storage. This information is managed in dedicated sample databases, which complement data catalogs and ELNs.

Sample databases allow researchers to record detailed attributes, such as composition, state of matter and storage conditions, and link these attributes to datasets collected at different instruments or facilities. Integration between sample databases and metadata catalogs ensures that provenance is maintained and that scientists can trace results back to the original sample context. PIDs such as IGSNs play a key role in this process, providing stable references that connect samples to datasets and publications.

Interest in sample databases is growing across the PaN community. Some institutions, such as HZB and HZDR, implement dedicated solutions, while others, including FAU and FZJ, are exploring options such as NOMAD Oasis or SampleDB for broader adoption. Kiel University operates an IGSN minting service, which could serve as a foundation for integrating sample identifiers into catalogs.

A coherent strategy for sample databases will be essential for enabling data reuse and supporting FAIR principles. By linking samples, datasets and publications through PIDs, the PaN community can ensure that experimental context is preserved and accessible for future research.

### 4.6. Lessons learned

Experience across the PaN community has highlighted several important lessons for the design and deployment of metadata catalogs.

Schema flexibility is a key distinction between the two systems. SciCat’s document-based model adapts easily to diverse experiments, while ICAT’s relational structure enforces strict consistency. Flexibility supports evolving workflows but requires governance to prevent inconsistencies; rigidity streamlines querying and integration but limits handling of unforeseen metadata.

Another lesson relates to metadata enrichment and ingestion workflows. Raw-data files are immutable and sometimes even inaccessible once archived, making it essential for catalogs to allow updates and additions after ingestion. This capability supports corrections and quality annotations without altering the original data. Automated processes can streamline metadata extraction, but manual curation remains necessary for high-quality datasets, as demonstrated by projects like RefXAS (Paripsa *et al.*, 2024 ▸) (see below).

Defining the granularity of datasets is also critical. Practices vary from treating individual scans as separate datasets to grouping them into logical units. Automated ingestion ensures completeness, but curated datasets provide greater scientific value and usability. Balancing these approaches requires clear policies and collaboration between instrument scientists and data managers.

Finally, adoption trends show that building critical mass is vital for sustainability. SciCat has attracted strong community support, with deployments at DESY, MLZ and HZDR. International facilities such as ESRF, ISIS, Diamond and ALBA contribute to an ecosystem around ICAT, reinforcing the need for interoperability and shared standards. These experiences underline that technical solutions alone are not enough; community engagement and harmonized practices are essential for success.

## 5. Case studies

Examples include HZB’s ICAT-based system for beamline data, DESY’s public data portal built on SciCat, and the RefXAS database for X-ray absorption spectroscopy (XAS). These cases demonstrate the importance of schema design, metadata quality, and integration with PIDs.

### 5.1. HZB catalog of raw data and data publications

HZB uses the ICAT metadata catalog for research data management and to implement the HZB Data Policy (https://hz-b.de/datapolicy). It is the central information hub that many workflows build upon. The metadata in the catalog are organized in terms of Investigations, Datasets and Datafiles. The Investigation defines the scientific context: why have the data been collected? Most, but not all, investigation in ICAT corresponds to a proposal from the HZB user office portal GATE. Access permission to the data is mostly defined at the investigation level. The Dataset defines the context of the creation of the data: how have the data been collected? In general, a dataset corresponds to an individual measurement at one of HZB’s instruments. Experimental parameters are stored in the metadata catalog at the level of the dataset. In general, the dataset is the smallest logical unit of data that is individually referenced and is attributed a PID.

The storage for the data is managed by ICAT. For archival, data are stored on tape. ICAT automatically triggers the restoration of the data to disk upon request. HZB provides the creation of data publications as a separate service. The bibliographic metadata are manually reviewed and curated, the data publication gets its own dedicated landing page and a DOI is registered. This makes the data publication fully citable. In most cases, a data publication is requested for the data that support a journal article. So it is not the raw data but a new dedicated dataset that is the subject of the publication.

PIDs are used wherever it makes sense. Raw data are attributed ePIC handles (https://www.pidconsortium.net/) for all investigations and datasets. The reason for not using DOIs here is that raw datasets are collected in such numbers that a manual curation of the metadata is not feasible. The metadata are constrained to what can be collected automatically. As a result, one obtains rather high quality in the physical parameters of the measurement but only poor bibliographic metadata that do not reach the quality threshold that one would expect for a DOI. Instruments are attributed DOIs to identify them. The HZB sample database SEPIA is under development and will be able to register IGSNs for samples. ORCIDs and RORs are used to identify researchers and organizations in the metadata.

The HZB ICAT provides an OAI-PMH endpoint that is harvested regularly by B2FIND to disseminate the metadata for raw data and data publications.

### 5.2. Public data catalog

The primary goal of establishing the portal (https://public-data.desy.de) was to provide a metadata catalog for high-quality processed, analyzed and published PaN data. The service is designed to address the needs of both data users and data owners, while promoting FAIR data principles and best practices in research data management.

The portal leverages DESY’s high-performance storage infrastructure dCache (Schwank *et al.*, 2015 ▸) for long-term preservation and provides access via standard protocols (HTTPS, WebDAV, NFS). Metadata follow community standards, including NeXus/HDF5 for scientific data and schema extensions for domain-specific techniques such as reflectometry.

For data owners, it offers a convenient and streamlined workflow for uploading and publishing high-quality PaN data, to ensure that data underlying publications are findable, citable and reproducible, or to fulfill open-data requirements from funders or journals. It enables the assignment of DOIs, allowing datasets to be identified and cited persistently.

For data users, it provides access to high-quality well curated PaN data; offers rich and standardized metadata, enabling users to find, understand and reuse datasets effectively; and enables reuse of datasets to understand and reproduce published research, for new scientific studies, method development or machine learning model training.

To complement the catalog, a dedicated upload tool (https://sisyphos.desy.de/) was developed. This was necessary as publishing experimental PaN data is a multi-step process involving standardization, validation and curation. In addition, direct metadata upload with SciCat requires either the submission of a correctly formatted JSON file that follows the SciCat data model, or the use of a limited metadata entry interface, making it unsuitable for most data owners.

Sisyphos is built around a metadata schema that reflects community conventions. The schema is expressed in LinkML and serves as the single source of truth for metadata structure and semantics. First focusing on reflectometry data, we started with a metadata schema based on the ORSO format from the Open Reflectometry Standards Organization. This approach proved highly effective: the LinkML schema is used to automatically generate metadata input forms (implemented via Streamlit). This allows us to easily extend the upload workflow to updates of the schema or other experimental techniques by just replacing the underlying metadata schema.

An additional benefit is that the metadata following the LinkML schema can be systematically translated into a representation compatible with the SciCat data model, acting as a bridge between domain-specific community standards and the catalog’s internal schema. By relying on LinkML, Sisyphos can also validate user-provided metadata directly against the schema. Schema-based validation significantly improves metadata quality before datasets are uploaded and reduces the burden on curators later in the process.

At present, curation in Sisyphos is manual, involving the inspection of both data files and metadata to assess completeness and correctness before publication. While this approach can ensure high quality, it comes with significant drawbacks. Manual review is time-consuming and cannot be sustained with limited staff once dataset numbers increase. Users may experience substantial waiting times before their datasets become publicly available, reducing motivation to publish data. Manual inspection increases the risk of overlooked issues. Without formalized criteria, curation outcomes may vary between curators and over time.

Publishing data requires additional effort from data owners, often with limited immediate personal benefit. As a result, the upload process must be highly user-friendly and the number of requested metadata entries must strike the right balance between relevance and effort. In practice, the users who actively upload datasets fall into two main groups: (*a*) researchers who need citable datasets to accompany scientific publications and (*b*) users who intentionally upload datasets for reuse, for example as training data for machine learning applications. For broader adoption, lowering the entry barrier while preserving metadata quality remains a central design challenge.

### 5.3. RefXAS: a curated reference database for X-rayabsorption spectroscopy

The RefXAS project (Gaur *et al.*, 2023 ▸; Paripsa *et al.*, 2024 ▸, 2025 ▸) (http://xafsdb.ddns.net/), developed under DAPHNE­4NFDI in collaboration between BUW, KIT, TUB and DESY, aims to establish a comprehensive reference database for XAS. The database focuses on both raw and processed data from X-ray absorption near-edge structure and extended X-ray absorption fine structure measurements of functional materials, with plans to include X-ray emission spectroscopy in a later stage.

A key feature of RefXAS is its user-friendly submission workflow. Researchers can upload datasets together with metadata via a dedicated web interface. Metadata are ingested into SciCat, while data files are stored in object storage. Automated query capabilities and visualization tools allow users to explore and assess the data efficiently.

To ensure reusability, the project defined a set of metadata fields specific to XAS experiments, covering sample details, bibliographic information, spectra and instrument parameters. Automated quality checks, such as edge step, energy resolution and signal-to-noise ratio, are applied during ingestion, providing immediate feedback to contributors and supporting curators in maintaining high data quality.

The curated nature of RefXAS benefits both contributors and users. Researchers can compare spectra for identical samples measured at different facilities, enabling studies of instrument-dependent effects. Automated preprocessing and quality control follow established protocols, ensuring consistency and reliability. By combining automated workflows with manual curation, RefXAS demonstrates how specialized databases can enhance data reuse and foster collaboration within the PaN community.

## 6. Recommendations

Based on the experiences gathered in DAPHNE4NFDI and discussions across the PaN community, we outline the following recommendations to guide the development of metadata catalogs.

### 6.1. Consolidation

The current landscape includes multiple catalog solutions, reflecting diverse institutional needs. Complete unification is unrealistic, but minimizing further fragmentation is essential. SciCat is recommended as the primary solution for new deployments.

### 6.2. Quality

First and foremost, the quality of the data in the database must be clear. The users must be aware if they are reviewing raw/reduced data from an experiment, processed data or high-quality reference datasets.

### 6.3. Interoperability

Cross-facility data discovery requires common APIs and metadata standards (Lohstroh *et al.*, 2024 ▸). We recommend implementing PaNOSC endpoints and OAI-PMH interfaces across all participating catalogs to enable federated search and metadata harvesting. A core metadata set for indexing should be defined, with optional extensions for domain-specific needs. Catalog systems should expose consistent REST APIs for ingestion, search and export, and provide commonly used plugins for systems used in DAPHNE4NFDI. PIDs must follow a unified strategy: DOIs for curated datasets, IGSNs for samples and ORCIDs for researchers.

### 6.4. Metadata enrichment

Catalogs should support post-ingestion metadata updates. Raw-data files are immutable but workflows often require corrections (*e.g.* typos in sample names) or additions (*e.g.* quality indicators). Catalog-level editing capabilities should be implemented with safeguards to maintain data integrity. Relationships between raw, processed and published data must be preserved. Automated quality metrics and manual curation options should be included to help users assess data reliability.

### 6.5. Integration with sample databases and ELNs

Linking catalogs with sample databases and ELNs is essential for providing experimental context and enabling reuse. Persistent sample identifiers such as IGSNs should be adopted to ensure global interoperability. Clear separation of responsibilities between catalogs, sample databases and ELNs will be necessary for efficient workflows.

### 6.6. AAI harmonization

Authentication and authorization systems vary widely across institutions, creating fragmentation. We recommend standardizing on Helmholtz ID, based on the Authentication and Authorization for Research and Collaboration (AARC) initiative, as the primary federated identity provider for PaN facilities, while supporting external providers such as umbrellaID, ORCID and eduGAIN for international collaboration. A common role model (*e.g.* administrator, instrument scientist, data curator, external user) should be defined and enforced across catalogs. Mapping between proposal systems and federated IDs will be required to ensure continuity as scientists move between institutions.

## 7. Conclusions

Metadata catalogs have become indispensable for implementing FAIR principles in PaN science. They enable data to be findable, accessible, interoperable and reusable across facilities and research institutions. While multiple catalog systems will continue to coexist, interoperability and shared standards are essential to ensure that this diversity does not hinder data discovery and reuse.

Several key observations emerged during DAPHNE­4NFDI. First, most participating institutions began without a metadata catalog, highlighting the scale of progress achieved. Second, fragmentation of software solutions is unavoidable due to legacy systems and institutional requirements, making interoperability a central challenge. Third, compatibility with European PaN facilities remains a priority for the German user community, reinforcing the need for alignment with international standards.

Consolidation efforts should aim to minimize further fragmentation. SciCat is recommended as the primary solution for new deployments within DAPHNE4NFDI, while other systems such as ICAT, SampleDB, NOMAD and Invenio continue to play important roles in specific contexts. Regardless of the chosen system, interfaces should be harmonized so that users experience a consistent search environment and federated access across catalogs.

Semantic technologies such as SPARQL and knowledge graphs will play a key role in connecting heterogeneous catalogs. Representing metadata as linked data and exposing it through SPARQL endpoints will enable uniform querying across systems. Knowledge graphs can integrate diverse schemas and vocabularies, supporting richer queries and reasoning over distributed datasets, in particular when supported by large language models. This approach aligns with FAIR principles by improving findability, interoperability and reusability across infrastructures.

Looking ahead, the PaN community must collaborate to build a federated FAIR-compliant catalog infrastructure that serves both facility and university researchers. This will require not only technical solutions but also coordinated policies for metadata standards, PIDs and authentication. By addressing these challenges collectively, we can ensure that PaN data remain a valuable resource for science well into the future.

## 8. Declaration of generative AI and AI-assisted technologies in the writing process

During the preparation of this work, the authors used ChatGPT in order to improve the quality of writing. After using this tool, the authors reviewed and edited the content as needed and take full responsibility for the content of the published article.

## Figures and Tables

**Table 1:** Data-management solutions by institution GAU, EKU and TUB are abbreviations for Georg-August University of Göttingen, Eberhard Karls University of Tübingen and Technical University of Berlin, respectively.

Institution	Own raw data	Group data	Public data	Comment
DESY	SciCat†	SciCat‡	SciCat†	§
HIFIS at DESY	N/A	N/A	SciCat†	–
CAU	SciCat†	SciCat†	Homebrew	–
TUM	SciCat†	None	None	–
KIT	SciCat†	None	None	¶
FAU	Catacore	OpenBIS††	SciCat†	–
	NOMAD	NOMAD	–	–
Uni Siegen	SciCat†	None	None	–
TUB	SciCat†	None	None	–
	eLabFTW (https://www.elabftw.net/)	–	–	–
BUW	SciCat†	None	None	–
GAU	None	None	None	–
LMU	SciCat†	None	None	–
EKU	None	SciCat†	None	–
RWTH	None	None	None	–
EuXFEL	myMdC	None	myMdC‡‡	§§
FZJ	SampleDB	SampleDB	SampleDB	–
	SciCat†	–	–	–
Hereon	SciCat†	None	None	–
MLZ	SciCat†	None	None	–
EMBL	ISPyB	None	None	§§
HZB	ICAT	None	ICAT	–
HZDR	SciCat†	None	Invenio	–
ESRF	ICAT	None	ICAT	–
Diamond	ICAT	None	ICAT	–
ISIS	ICAT	None	ICAT	–
ALBA	ICAT	None	ICAT	–
SESAME	ICAT	None	ICAT	–
Sirius	ICAT	None	ICAT	–
ESS	SciCat	None	SciCat	–
PSI	SciCat	None	SciCat	–
ILL	Homebrew	None	Homebrew	¶¶

† Decision taken, currently being integrated into facility.

‡ Several individual instances.

§ https://public-data.desy.de, https://fsdata.desy.de/.

¶ Some data are published on KIT open LabIMotion.

†† Use of OpenBIS mandated by running SFBs.

‡‡ myMdC includes public raw data – the existing entries are automatically flagged as open once a proposal gets beyond the embargo period. An integrated mechanism (like Globus) to easily find/access open data is not yet available.

§§ Extremely specialized database.

¶¶ Legacy software.

## Data Availability

No datasets were reported in this study. Accordingly, there are no data available within the article.
